# 喉罩通气全麻支气管镜下高频电灼联合冷冻治疗气管错构瘤2例并文献复习

**DOI:** 10.3779/j.issn.1009-3419.2011.02.14

**Published:** 2011-02-20

**Authors:** 继旺 王, 茂 黄, 栩 齐, 梅梅 李, 林福 周, 凯生 殷

**Affiliations:** 210029 南京，南京医科大学第一附属医院呼吸内科 Department of Respiratory Medicine, the First Affilliated Hospital, Nanjing Medical University, Nanjing 210029, China

气管狭窄有多种病因，其中气管错钩瘤是其原因之一。气管错构瘤属于腔内型错构瘤，在临床上比较少见。传统的治疗方法是外科手术切除。近10年来，随着内窥镜介入治疗技术日臻成熟，为气管狭窄的治疗提供了新的方法。本文通过分析南京医科大学第一附属医院呼吸科收治的2例气管错构瘤，并复习国内相关文献，旨在提高对气管错构瘤介入治疗的认识。

## 1 临床资料

患者1，男，51岁，因“反复咳嗽10年，加重7 d”于2009年9月入院。咳嗽以刺激性干咳为主，常年阵发性发作，无明显季节性。入院前胸部CT检查示：气管右后侧壁见一椭圆形脂肪密度影，CT值99 Hu，密度均匀，边界清晰（图 1A）。入院后电子支气管镜检查发现声门下7 cm处有一1.5 cm×2.0 cm大小的新生肿物（图 1B）。基底部位于气管右侧壁，宽蒂，淡黄色，表面凸凹不平，稍有分叶，表面粘膜覆盖，血管清晰，周围粘膜光滑无浸润。管腔阻塞达75%以上，活检病理证实为气管错构瘤（图 1C）。遂进行了喉罩（欧普乐牌，台湾旭邦工业有限公司）全麻下支气管镜介入治疗。介入手术过程：患者仰卧位，左下肢紧贴皮肤放置内衬盐水纱布的电极板。麻醉诱导前，静注阿托品0.5 mg、地塞米松10 mg以减少呼吸道分泌物并预防呼吸道粘膜水肿。在心电、脉氧及血压监测下，静脉推注咪唑安定2 mg、芬太尼50 μg、丙泊酚50 mg麻醉诱导后顺利插入4#喉罩，喉罩与呼吸机用三通连接管连接。丙泊酚、雷米芬太尼泵注维持麻醉。电子支气管镜（BF260型，日本Olympus公司）由三通连接管带有密封帽端口进入，到达病灶后将圈套器或电灼探头经活检孔送入气管病灶处，当视野内看到绿色标记时，打开电切电凝电源开关，将高频电治疗仪（PSD30型，日本Olympus公司）电切的输出功率定为30 W-35 W，电凝输出功率为35 W-40 W。首先将圈套器（SD-18c-1、SD-7c-1，日本Olympus公司）环绕肿物，助手拉紧收缩圈套器，足踏开关5 s-10 s，切除的组织块用异物钳取出。因基底部较大，不能进行套切，换用电极探头置于病灶表面，通电1 s-2 s，多次点击电灼，使病灶凝固、汽化（图 1D），坏死物质通过活检钳或吸引及时清除。当基底部接近管壁时，换用冷冻治疗机（K300型，北京库蓝）实施冷冻，每个冷冻点持续2 min-4 min。1周后进行支气管镜检查并清理坏死组织。随访8个月未见复发及并发症出现。

**1:**
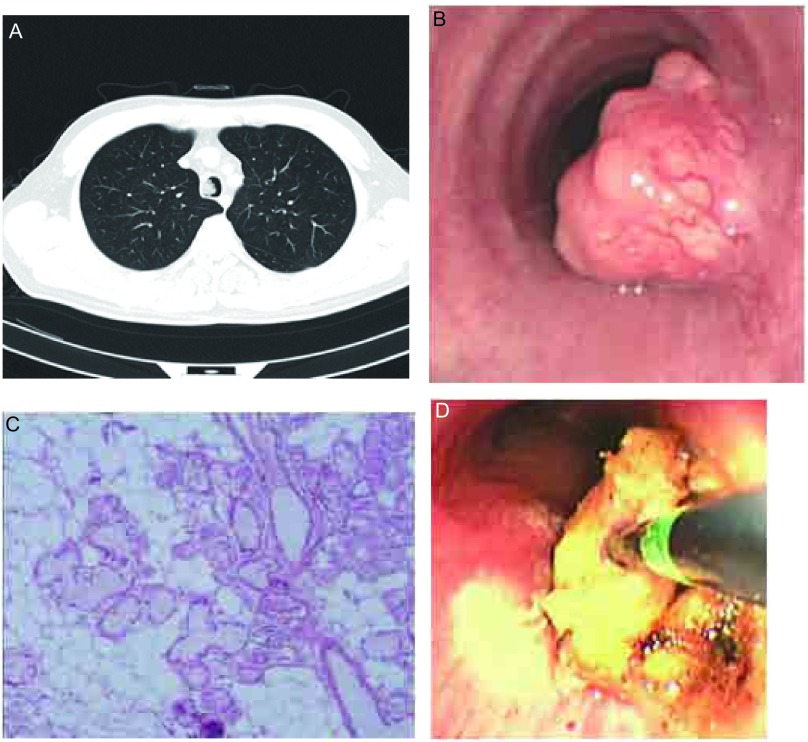
患者1的胸部CT、电子支气管镜下气管错够瘤的表现及病理结果。A：一软组织影位于气管腔内，表面略有分叶，基底部位于右侧壁；B：管腔内椭圆形肿块，表面凸凹不平，略有分叶，基底宽，管腔阻塞75%以上；C：脂肪组织及混合性腺体增生（HE, ×200）；D：高频圆钝探头置于病灶表面进行电灼。 The appearance of tracheal hamartoma with chest CT, bronchoscopy and pathological findings in case 1. A: The chest computed tomogram showing a lobulated soft tissue mass that attached to the right lateral part of the trachea; B: A oval lobulated mass with a wide sessile base occluding 75% of the lumen. C: Components shown in detail are adipoid and mesenchymal tissue (HE, ×200); D: Using electrocautery the blunt probe is applied to granulation tracheal hamartoma.

患者2，男，68岁，因“胸部不适，行胸部CT检查发现气管内肿块影”于2009年11月入院。患者无明显咳嗽、咳痰症状，无吸烟史。查体无阳性体征。胸部CT显示：气管内见一圆形低密度影，与气管右侧壁相连，CT值为144 Hu（图 2A）。患者入院后常规支气管镜检查显示：气管下段距隆突4 cm处见一圆形肿物向腔内生长，质韧，触之不易出血，肿物表面光滑，宽蒂，黄白色，管腔阻塞约35%左右（图 2B）。给予针吸活检，见黄色油状物，考虑为良性病变。择期在喉罩通气全麻电子支气管镜下进行圈套器套切后切除腔内肿物。异物钳取出（图 2C），病理结果为气管错构瘤（图 2D）。残基部（图 2E）用冷冻探头进行多点冷冻治疗后基本消除。术后给予雾化吸入普米克令舒以减轻气道水肿。随防半年未见复发。

**2:**
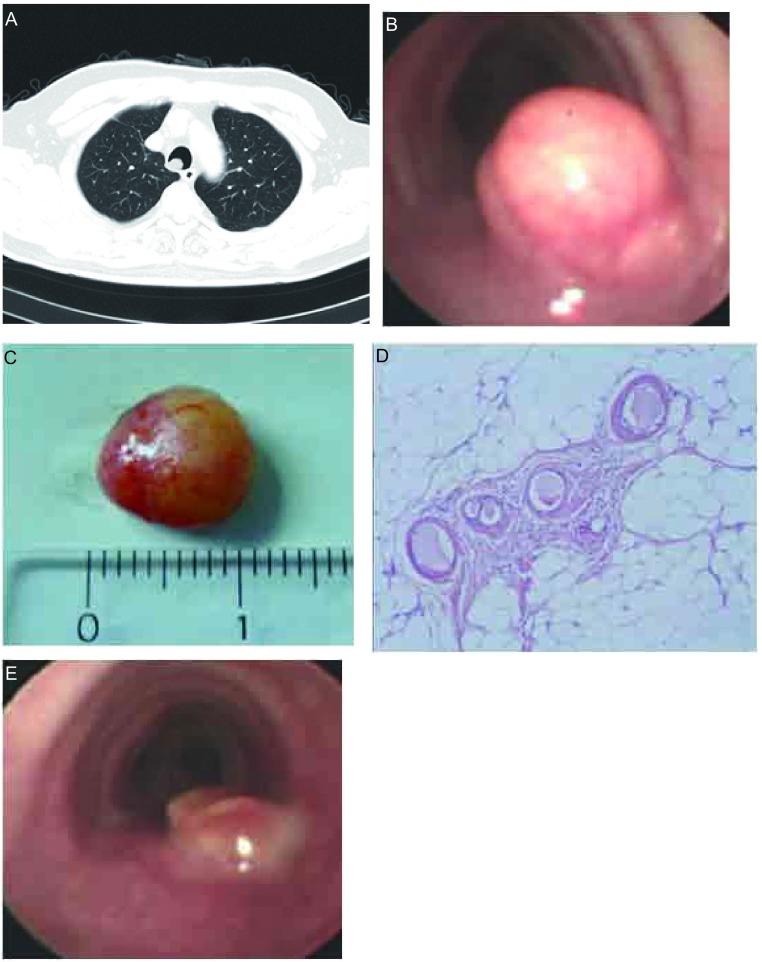
患者2的胸部CT、电子支气管镜下气管错够瘤的表现及病理结果。A：胸部CT显示气管腔内一圆形肿物，基底部位于气管右后侧壁，宽蒂，表面光滑；B：气管腔内见圆形新生物，表面光滑，宽蒂；C：应用圈套器切除的气管错构瘤，红黄色，表面光滑，大小为1.0 cm×0.8 cm；D：脂肪组织及少许黏膜腺体（HE, ×200）；E：气管错构瘤经电圈套器套切后残根部情况。 The appearance of tracheal hamartoma with chest CT, bronchoscopy and pathological findings in case 2. A: CT scan demonstrated a smooth surfaced round mass arising from the right anterolateral wall of the trachea with a wide sessile base in case 2; B: A yellow, smooth shining surfaced with a wide sessile base polypoid lesion just above the back wall of the trachea; C: The yellowish-red, smooth shining surface tracheal hamartoma removed by electrosurgical snare in case 2, the mass measured 1.0 cm×0.8 cm; D: Histologic findings with mature fat cells and mucinous glands, and respiratory epithelium (HE, ×200); E: The view of tracheal lumen after tracheal hamartoma removed by electrosurgical snare.

## 2 讨论

以“气管错构瘤”、“管内型错构瘤”为检索词，在中国知网、中国生物文献数据库检索近15年来的国内文献，检索到经病理确诊的成年气管错构瘤病例6例（表 1），加上本院2例共8例。平均年龄为（49±14）岁，临床上以呼吸困难为主要症状，多误诊为“支气管哮喘”、“慢性支气管炎”，误诊时间较长。病灶可发生在气管的任何部位。支气管镜下特征：圆形肿块、表面光滑，宽蒂多见，无周围粘膜浸润，就诊时肿块阻塞管腔多在50%以上，较少能早期发现，例2患者仅在常规胸部CT检查时被发现。胸部CT检查对气管错构瘤的诊断有重要的价值。其特点是边界清晰光滑，局部富含脂肪组织，或脂肪组织存在钙化灶^[1]^。当CT显示肿物含有较多脂肪组织时，则高度提示气管错构瘤的诊断，CT显示肿块内有脂肪组织或爆米花样钙化时更有诊断意义^[2]^。而通过支气管镜检查并进行活检则可明确诊断。

**1:** 6例气管错构病患者的临床资料及治疗 Summary of the published cases of tracheal hamartom

Year	Age (yr)	Sex	Misdiagnosis time	Symptom	Location of the lesion	Shape of the mass	Gross	Treatment
1996	57	Male	1	Cough	Upper 1/3	Oval	> 0%	Flexible bronchoscopic recetion
1996	34	Female	1	Hoarseness, dyspnea	Upper 1/3	Round	> 75%	Death
1998	31	Female	12	Wheezing	Upper 1/3	Round	> 90%	Operation
2003	54	Male	24	Dyspnea	Upper 1/3	Round	> 75%	Operation
2004	35	Female	36	Dyspnea	Lower 1/3	Round	> 75%	Operation
2004	65	Male	48	Dyspnea	Middle 1/3	Round	> 75%	Operation

随着呼吸介入技术的进展及仪器设备的更新，对气道肿瘤尤其是良性肿瘤的介入治疗已取得了令人瞩目的进展。以往很多需要手术治疗或根本无法治疗的一些气道腔内病变，均可以借助纤维支气管镜介入治疗而获得临床治愈^[3]^。因此，经气管镜腔内治疗已成为气管内病变的主要治疗方法^[4]^。

支气管镜下高频电切和电凝、套切等作为一种介入治疗方法已广泛应用于腔内狭窄的治疗^[5]^。若选择在局麻下行支气管镜介入治疗，由于反复电切和电凝刺激及频繁咳嗽，血氧分压下降，存在窒息的风险。局麻下患者出现呛咳及躁动，从而影响医生对病变的仔细观察及电切、套切及电凝病灶的准确性，易导致治疗失败或出现并发症，因此多选择在全身麻醉建立人工气道方式下进行介入治疗。

喉罩通气全麻在呼吸介入治疗中优势明显。喉罩是一种新型的通气方式，可在全身麻醉中保障患者呼吸道通畅。放入咽喉后能与喉形成一个密封圈，既可让患者自主呼吸，又可进行正压通气，是介于气管导管与面罩的通气工具，与气管插管比较，喉罩操作简便，易掌握，不需要特殊器械。喉罩的置入对咽喉和气管不产生机械损伤，对血液循环影响轻微。同时采用喉罩建立人工气道实施全身麻醉，不需要占据患者的气道内空间，即使存在气道病变，亦不会造成明显的阻碍，因而可为术者提供足够的操作空间，并保证患者的通气和氧合功能。另外，喉罩是按照人体解剖形态制作而成，不进入气管内，术后患者较易耐受，异物感少，腺体因刺激减少，分泌减少，同时纤毛活动没有受到影响，从而减少了术后肺部感染的机会。通过喉罩可以反复插入进行支气管镜治疗，有效控制呼吸，改善缺氧状态，并可根据狭窄的程度、范围而决定治疗的时间，能够满足足够的介入治疗时间需求，达到最佳疗效，提高了镜下治疗的安全性。通过套切切除瘤体而不能取出瘤体时，可同时将喉罩、支气管镜及瘤体一同拨出，故介入治疗简便而安全。

喉罩通气全麻是目前进行气管内介入治疗较理想的麻醉方法。而对于气管狭窄，尤其是上段狭窄（声门下5 cm内）的呼吸困难患者，喉罩通气全麻是目前唯一有效控制气道的方法^[6-8]^。

近15年来国内对气管错构瘤的治疗，仍以传统手术方式为主，本文报道的2例支气管镜下介入治疗，取得了很好的疗效。

气道的介入治疗方法有多种。常用的治疗技术主要包括激光、高频电、氩等离子、冷冻、微波等。其中激光、高频电、氩等离子是常用的治疗手段。而高频电灼、套切作为一种物理治疗手段，通过高频电流聚集于电极、电圈所接触的组织，使组织发生凝固及坏死，治疗范围较易控制，不易引起气道壁的穿孔和大出血，且成本低^[9]^，较适合我国国情。本文正是应用高频电切、电凝原理成功对2例气管错构瘤进行了切除并对基底部进行了冷冻治疗。1例已随访8个月，另外1例随访半年，均未发现复发及并发症，取得了良好的效果。

对于气管错构瘤经过由套切、电凝等治疗后的残余组织的处理，目前报道的方法不一^[10]^。部分研究者对残留的基底部组织不做任何处理，但尽量用电凝、电切等方法减少残留组织。但由于基底部组织与管壁相连，且厚度较薄，稍有不慎会损伤正常管壁及粘膜，严重时会造成气管壁穿孔、气胸、纵隔气肿及大出血，因此要慎之又慎。尽管目前未见有关穿孔的报道^[9]^，但动物实验^[11]^已证实有导致穿孔、坏死及软骨损伤的风险。研究者^[12]^应用圆钝电极在30 W功率时对6例即将切除的支气管进行1 s-5 s的电凝实验，手术切除后经组织病理分析发现，当电切时间＞3 s时，管壁即出现坏死、溃疡及软骨损伤。而国外有研究者^[13]^应用冷冻方法对支气管错构瘤残基组织进行治疗，取得了良好的效果。本文对2例经套切、电凝治疗后剩余的基底部组织进行冷冻治疗，随访结果表明治疗效果好，无并发症发生，且残余组织清除彻底，未见复发及其它异常。

冷冻治疗有明显的优越性。冷冻治疗安全性极高，不损伤气道软骨，气道穿孔危险性最小，治疗后不会引起管壁挛缩狭窄及其它并发症^[14]^。其作用原理是低温使细胞内产生结晶，细胞崩解，同时还可以使治疗区域内的小血管内皮细胞发生损伤，局部血栓形成，进而引起组织缺血坏死。其特点是对含水丰富的组织效果好^[15]^，而对含水量少的组织如软骨则几乎没有损伤，因此对气道组织冷冻治疗时不易损伤软骨组织，安全性极高^[13]^。

总之，喉罩通气全麻是介入治疗气管错构瘤的良好选择，尤其对于气管上段狭窄的介入治疗，喉罩通气全麻是目前最佳选择^[6, 7, 16]^。而喉罩全麻支气管镜下对气管错构瘤进行高频电套切、电灼治疗是一种良好选择，费用低、安全、有效^[17, 18]^，而消除残留的基底部组织，冷冻治疗不失为一种安全、有效的选择。
